# Morphological changes of Intervertebral Disc detectable by T2-weighted MRI and its correlation with curve severity in Adolescent Idiopathic Scoliosis

**DOI:** 10.1186/s12891-022-05561-w

**Published:** 2022-07-10

**Authors:** Kwong Hang Yeung, Gene Chi Wai Man, Min Deng, Tsz Ping Lam, Jack Chun Yiu Cheng, Ka Chi Chan, Winnie Chiu Wing Chu

**Affiliations:** 1 Department of Imaging and Interventional Radiology, Faculty of Medicine, The Prince of Wales Hospital, The Chinese University of Hong Kong, Shatin, Hong Kong SAR; 2 Department of Clinical Oncology, Tuen Mun Hospital, Tuen Mun, Hong Kong SAR; 3 Department of Orthopaedics and Traumatology, Faculty of Medicine, The Prince of Wales Hospital, The Chinese University of Hong Kong, Shatin, Hong Kong SAR

**Keywords:** Intervertebral disc, Nucleus pulposus, Volume, Vertebral wedging, Magnetic Resonance Imaging, T2-weighted, Adolescent idiopathic scoliosis, Healthy controls

## Abstract

**Background:**

Our previous studies found disproportionate anteroposterior vertebral size is associated with severity of the scoliotic curves in adolescent idiopathic scoliosis (AIS) patients. Subsequent studies showed wedging of vertebral bodies (VB) had less contribution than intervertebral discs (IVD) to the anterior–posterior vertebral column length discrepancy in severe-AIS. However, the exact morphological changes of IVD were not clearly defined. This study aimed to evaluate the morphological and pathological changes of IVD and VB in AIS girls and healthy female controls.

**Methods:**

This study included 33 age-matched female controls and 76 AIS girls with a right-sided thoracic curvature. Wedge angle, height ratio and distance ratio of VB and IVD were measured on the best midline coronal and sagittal planes from reformatted MRI spine. Volumes of VB, IVD and nucleus pulposus (NP) were also evaluated on volumetric images. One-way ANOVA with Bonferroni correction and Pearson correlation tests were used.

**Results:**

There was significant difference in wedge angle and height ratio of VB and IVD between AIS and controls. In severe-AIS, the position of NP was significantly shifted to the convexity when compared with non-severe AIS and controls. Whereas, the volume of IVD and NP in severe-AIS was found to be significantly smaller. In addition, Cobb angle was significantly correlated with wedge angle and height ratio, and inversely correlated with the volume of NP.

**Conclusions:**

In addition to wedging of VB and IVD, there was significantly reduced volume of IVD and NP in AIS patients with severe curve, insinuating the mechanical effect of scoliosis leads to a compression on both IVD and NP before significant disc desiccation occurs. We postulate that the compression of IVD and NP can contribute to curve progression in severe-AIS, these patients are more prone to disc degeneration in adulthood if no operative treatment is offered. Further longitudinal study on these parameters is still warranted.

## Background

Adolescent Idiopathic Scoliosis (AIS) is a complex disorder characterized by the three-dimensional (3D) spinal deformity in the coronal and sagittal planes with vertebral rotation in the transverse plane [1]. In general, the spinal deformity progresses rapidly during the adolescent growth spurt, affecting 2–3% of children worldwide [1]. This is the most common type of scoliosis worldwide that mainly affects girls [1]. Patients with progressive curvatures are associated with significant morbidities and disfiguration of body image, and hence, regular monitoring of scoliosis during growth is therefore important for treatment planning [1–3]. Despite decades of dedicated research, the etiopathogenesis of this classic orthopedic disorder remains uncertain, which is believed to be multifactorial [1].

Based on our earlier studies, there were abnormal anthropometric and relative anterior spinal growths in AIS, suggesting that AIS subjects might have a faster disproportionate growth due to endochondral ossification [2, 3]. In addition, the spinal cord to vertebral column ratios in AIS patients with severe curvature were found to be significantly reduced, which demonstrated asymmetric growth between the spinal column and the neural systems [4–7]. More recently, greater fatty infiltration was found in the concavity of paraspinal muscles in patients with severe-AIS group [8]. Whereas in these studies using computed tomography (CT) imaging, an increase in anterior length of spinal column was dominantly contributed by an increased height of intervertebral discs (IVD) [9, 10]. These studies imply an asymmetric growth during the progression of AIS.

Additionally, other biomechanical and biochemical studies suggested that there was a correlation between the spinal deformity and IVD [11–13]. There was a relationship between the wedging of intervertebral discs (IVD)/vertebral bodies (VB) and spinal rapid growth in AIS with curve progression [13–18]. Likewise, vertebral wedging was also found to be associated with Cobb angle [14–16, 19]. Based on the preliminary finding from Violas et al., which reported the consequences of scoliotic surgery in lumbar region for patients with idiopathic scoliosis, an increase of the nucleus pulposus (NP) volume relative to the IVD was found which might indicate rehydration of subjacent disc at postoperative stage [20]. However, most of these studies have limitation of lacking comparison with asymptomatic controls. Likewise, as most of these studies were using computed tomography (CT) for evaluation, the precision on detailing the morphological changes in soft tissue such as IVD was limited.

Herein, with the advantage of better soft tissue resolution using MRI, the morphological change of IVD and its relation toward curve severity in AIS are better elucidated. Using a cross-sectional study, we aimed to investigate the morphological change of IVD and VB in female AIS with a right-sided thoracic curve and asymptomatic controls, and to correlate these changes with curve severity using MRI.

## Methods

### Participants

**Fig. 1:**
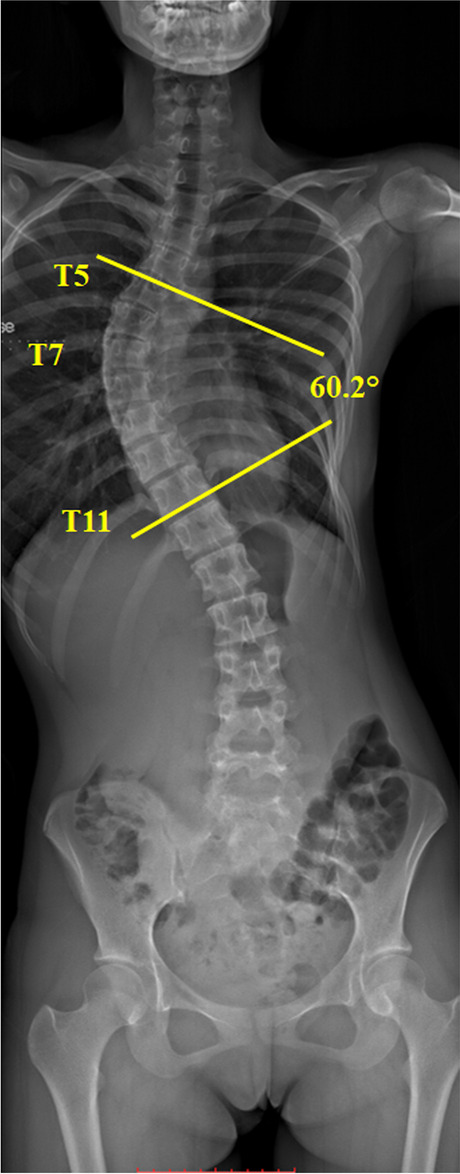
The Cobb angle measurement. Cobb angle is the vertebrae that are most tilted relative to the horizontal at upper and lower levels of each curve are measured; This scoliotic subject has a thoracic curve: upper end level = T5, apex = T7, lower end level = T11, convexity = right, Cobb angle = 60.2°

Participants with and without AIS were prospectively recruited during April 2006 to July 2010. Patients with AIS were recruited from our institutional scoliosis clinics, whereas the asymptomatic controls were volunteers from local schools. Like our previous studies, recruited participants must be Chinese female adolescents aged between 10–16 years old [8, 19]. The inclusion criteria for AIS patients was the presence of a major right-sided thoracic curvatures with the apex located between T7 to T9. Further, participants with conditions and medications such as bone remodeling affection, calcium metabolism, neuromuscular abnormalities, genetic diseases, chromosomal defects, autoimmune disorders, and endocrine disturbances would not be eligible for recruitment [8]. In addition, subjects have secondary scoliosis with known etiology such as neuromuscular scoliosis, congenital scoliosis, scoliosis associated with skeletal dysplasia and connective tissue abnormalities were excluded from this study [8]. The diagnosis of AIS with typical scoliotic curvatures was clinically examined using the Adam Forward Bend Test and radiographically using the standing whole spine posteroanterior radiographs by the institutional experienced orthopedic surgeons. Subjects with the initial Cobb angle of at least 10 degrees in radiographs was justified as the presence of scoliosis (Fig. 1). Whereas, all asymptomatic controls were also examined by experienced orthopedic surgeons to exclude the presence of scoliosis. All subjects and their parents were provided with written informed consent prior to joining this study. Ethical approval was obtained from our institutional Clinical Research Ethics Committee.

### MRI Examination

All asymptomatic controls and AIS subjects were acquired from a MRI examination and this examination was performed on a 1.5-T MR Scanner (Sonata, Siemens, Erlangen, Germany). The MRI machine contains an equipment of spine array coil [8]. Two main sequences were performed on the MRI scanning [8]. (1) Images of whole spine from foramen magnum to sacrum were acquired on sagittal plane using spin-echo T2-weighted sequence with TR = 6520/TE = 118 ms: 3 mm thickness with no gap, matrix = 256 × 152, field of view = 512 × 304 mm, pixel size = 1.5 × 1.5 mm [8]; (2) High resolution transverse (TS) image of the thoracic column (T1–T12) were acquired using spin-echo T2-weighted sequence with TR = 5730/TE = 93 ms: 2 mm thickness with no gap, matrix = 256 × 138, field of view = 172 × 256 mm, pixel size = 0.8 × 1.0 mm [8]. A new MRI examination had to be performed again when motion artifacts were spotted or removed if necessary.

### MRI morphological measurement

**Fig. 2:**
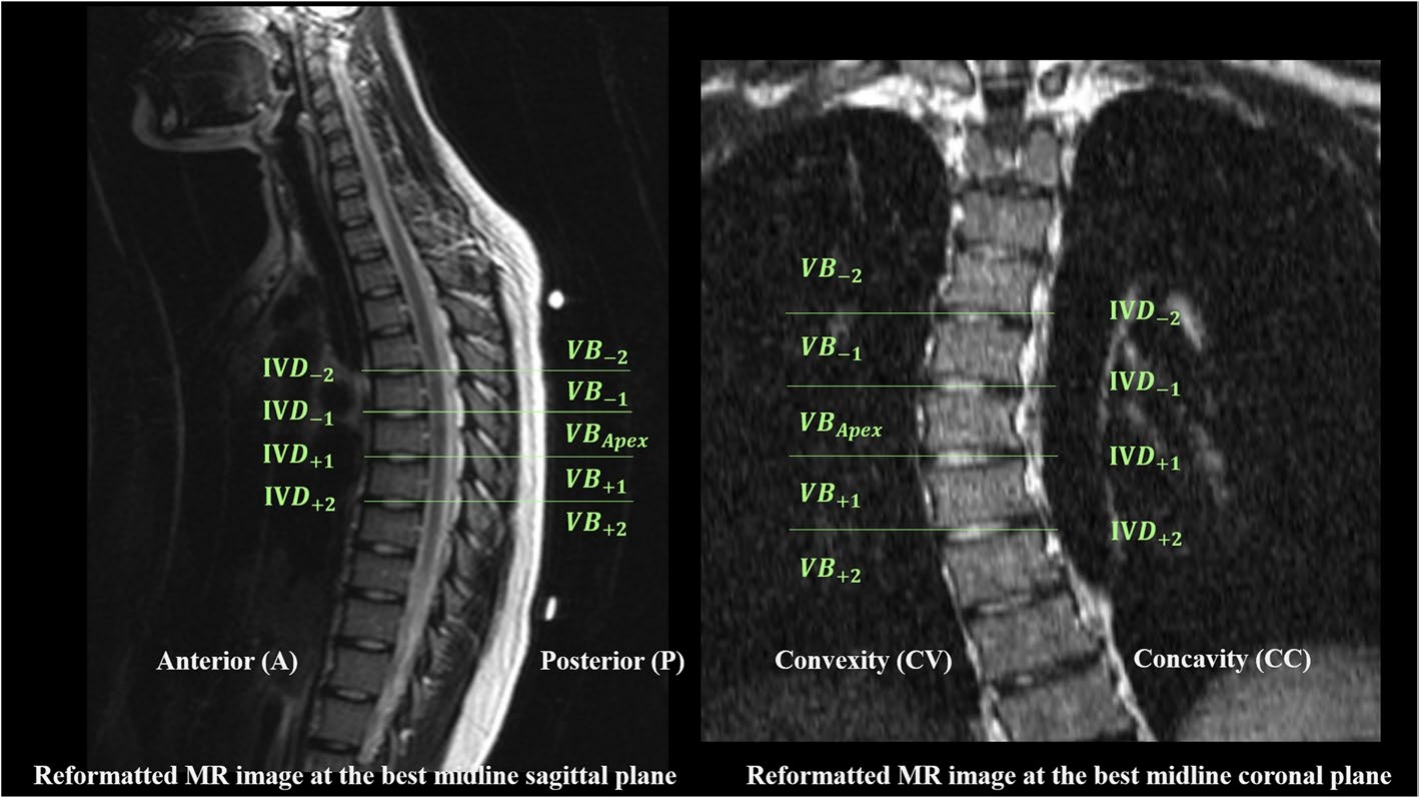
**A** T2-weighted MR reformatted at the best midline coronal and sagittal planes of a 15-year-old female AIS patients. The nomination of vertebrae (VB) and intervertebral discs (IVD) are shown

Philips DICOM Viewer (Philips, Best, Netherlands) was used for MR image tracing and measurement. In the 2D assessment, the sagittal MR images were reformatted along the centre of each vertebra to produce the best vertebral midline image on both sagittal and coronal planes (Fig. 2). In AIS subjects, measurements were taken along 5 VBs around the apex (including the apical vertebra, 2 vertebrae above and below) and 4 subjacent IVDs. For normal subjects, T8 vertebra was taken as the AIS-equivalent apical vertebra. Meanwhile T6, T7 and T9, T10 vertebrae were taken as the upper two and lower two vertebrae, respectively. 4 subjacent IVDs were also measured and compared to corresponding levels of AIS.

**Fig. 3:**
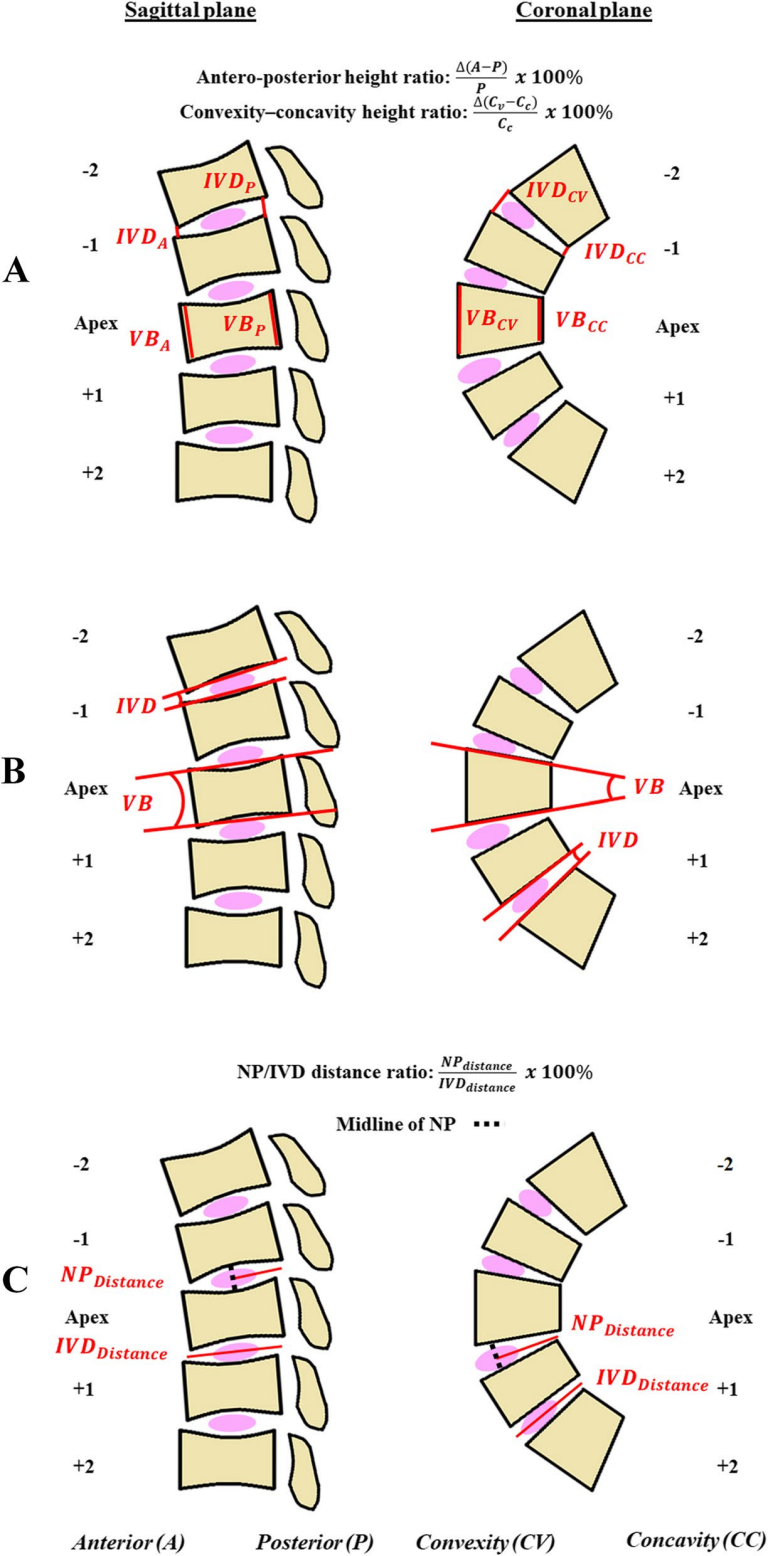
Schematic diagram showing measurements of **A** (**a**) anterior–posterior (AP) height ratio of both VB and IVD on sagittal plane and (**b**) convexity-concavity (${C}_{V}{C}_{C}$) height ratio of both VB and IVD on coronal plane. **B** Wedge angles (WA) of both VB and IVD on both sagittal and coronal planes. **C** Nucleus pulposus (NP) to IVD distance ratio

The measurement parameters are as follows (Fig. 3):

(1) Anterior–posterior (AP) height ratio of both VB and IVD on sagittal plane and illustrated in Fig. 3A [10, 21]. The AP height was the distance measured from the upper to lower border of either VB or IVD on the anterior and posterior margin respectively. Their ratios were calculated as $\frac{\Delta \left(A-P\right)}{P} \cdot 100\%$. When AP height ratio was positive (> 0) on sagittal plane, it indicated the vertebral/ disc height over the anterior margin was larger than that of the posterior margin, i.e. more wedging towards the posterior margin and vice versa.

(2) Convexity-concavity (${C}_{v}{C}_{c}$) height ratio of both VB and IVD on coronal plane and illustrated in Fig. 3A [22]. The ${C}_{v}$ or ${C}_{c}$ height was the distance measured from the upper and lower border of either VB or IVD on the convexity margin and concavity margin respectively and then their ratios calculated as $\frac{\Delta \left({C}_{v}-{C}_{c}\right)}{{C}_{v}} \cdot 100\%$. When ${C}_{v}{C}_{c}$ height ratio was positive (> 0) on the coronal plane, it indicated the vertebral/ disc height over the convex margin was larger than that of the concave margin, i.e. more wedging on the concavity side and vice versa.

(3) Wedge angles (WA) of both VB and IVD on both sagittal and coronal planes and illustrated in Fig. 3B. The wedge angle was the angle formed between the two tangential lines along the upper and lower border of either the VB or IVD. An increase in wedge angle on sagittal plane indicated the vertebral/disc height over the posterior margin was larger than that of the anterior margin, i.e. more wedging towards the anterior margin. An increase in wedge angle on coronal plane indicated the vertebral/ disc height over the convexity margin was larger than that of the concavity margin, i.e. more wedging towards the concavity margin.

(4) Nucleus pulposus (NP) to IVD distance ratio: Nucleus pulposus was defined as the T2 hyperintense centre of the IVD, while the IVD was defined as the entire intervertebral substance including the outer dark (T2-hypointense) margin and the bright (T2-hyperintense) center. The distance of NP was measured from the midpoint of the T2-hyperintense centre to either the posterior or concavity margin of the disc on sagittal plane and coronal plane, respectively. The distance of the IVD was measured along entire IVD from the anterior–posterior or convexity to concavity on sagittal and coronal plane, respectively. The NP to IVD distance ratio is between 0 to 1 and the formula is given $\frac{N{P}_{distance}}{IV{D}_{distance}} \cdot 100\%$ (Fig. 3C).

**Fig. 4:**
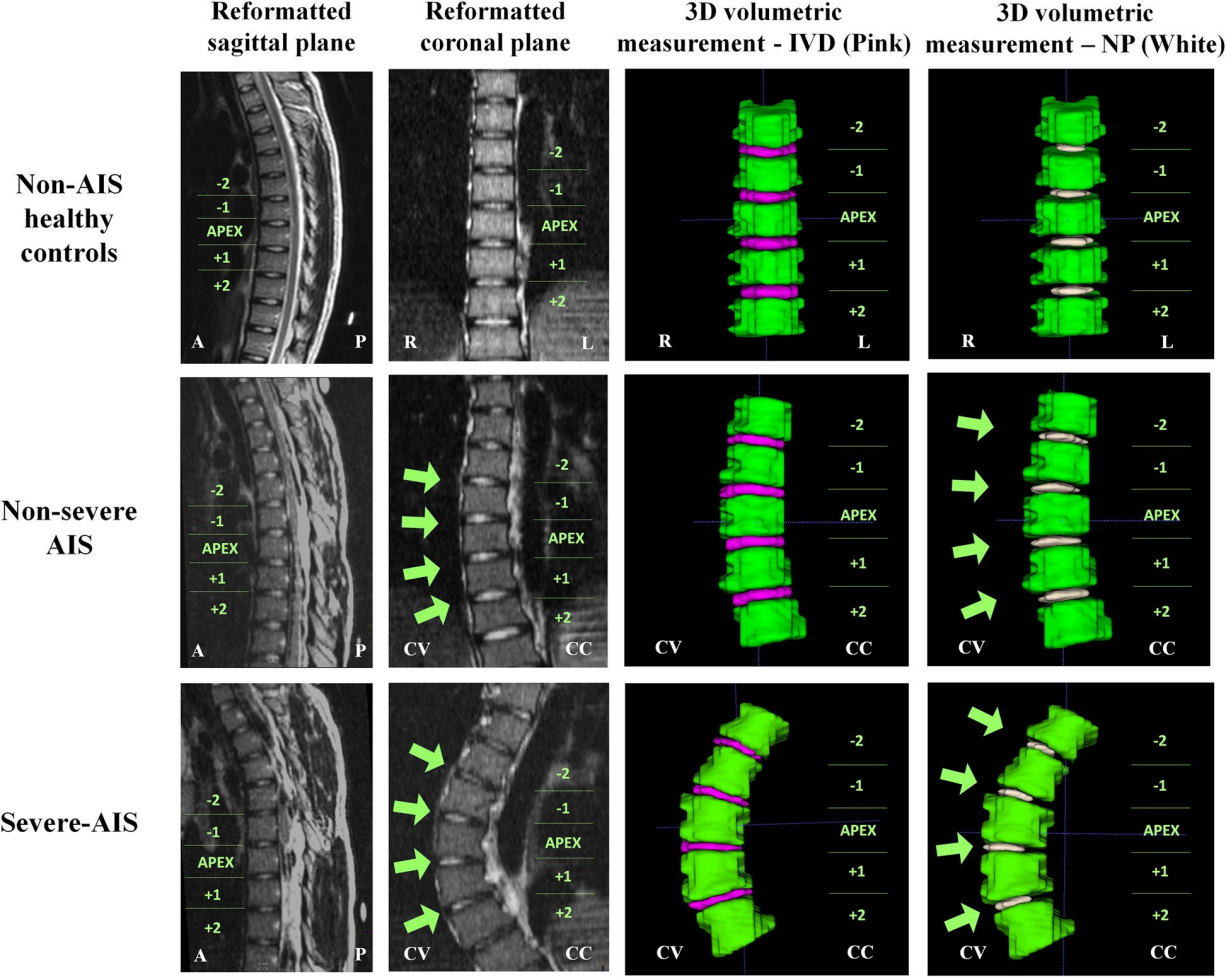
Representative 3D models showing volumetric assessment of VB, IVD and NP in normal controls, non-severe AIS and severe-AIS using manual segmentation and software of ITK-Snap. Note that the NP is shifted towards the convexity side in severe-AIS patient, which can be clearly seen as bright signal centre within the IVD on coronal reformatted image and as white coloured structure on the 3D volumetric image. IVD, intervertebral disc; NP, nucleus pulposus; A, anterior side; P, posterior side; CV, convex side; CC, concave side for all AIS subjects; R, right side; L, left side for all controls

The transverse MR images were used for volumetric assessment, the volume of VB, IVD and NP at the aforementioned levels of both normal controls and AIS were measured using manual segmentation and calculated by the software of ITK-Snap [23]. The method is illustrated in Fig. 4.

### Measurement Reliability

In this study, there were 10 subjects randomly chosen for the reliability of the image analysis in MRI. The analysis was then performed by 2 additional observers within an interval of 14 days for all parameters [8]. The subject information was blinded for each observer with the measurement being performed individually. Intraclass correlation coefficient (ICC) was used for accessing the intra- and inter-observer reliability measurement.

### Statistical analysis

All data was presented as mean ± standard error of mean. Analysis of variance (ANOVA) with Bonferonni correction was performed in group comparisons of demographic data and all MRI measurements. A Pearson correlation analysis was used for the correlations between MRI measurements and other variables. Statistical significance was considered when p-values was less than 0.05. The statistical analysis was calculated by the SPSS software (Version 25.0; SPSS, Chicago, IL, USA).

## Results

### Participant Characteristics

There were seventy-six patients with female AIS (mean age: 14.2 ± 0.2) with a dominant right thoracic curvature and thirty-three non-AIS female asymptomatic controls (mean age: 14.8 ± 0.3) were recruited. Demographic data of the subjects are shown in Table 1. No significant difference was found in the mean age in the demographic data, however, the coronal Cobb angle and BMI were significant different between each group, which was examined by two experienced orthopaedics surgeons (J.C.Y.C. and T.P.L.). Significant lower BMI was found in AIS group. The AIS subjects were then split into 2 groups according to the severity of their scoliotic curve based on the Cobb angle, non-severe AIS (*n* = 32, Cobb angle = 11°–30°) and severe-AIS (*n* = 44, Cobb angle = 45°–90°).

The result showed a reliable measurement method for the intra- and inter-observer agreement in MRI measurements of all parameters with ICC > 0.82.

### Vertebral bodies (VB) measurement analysis

**Fig. 5:**
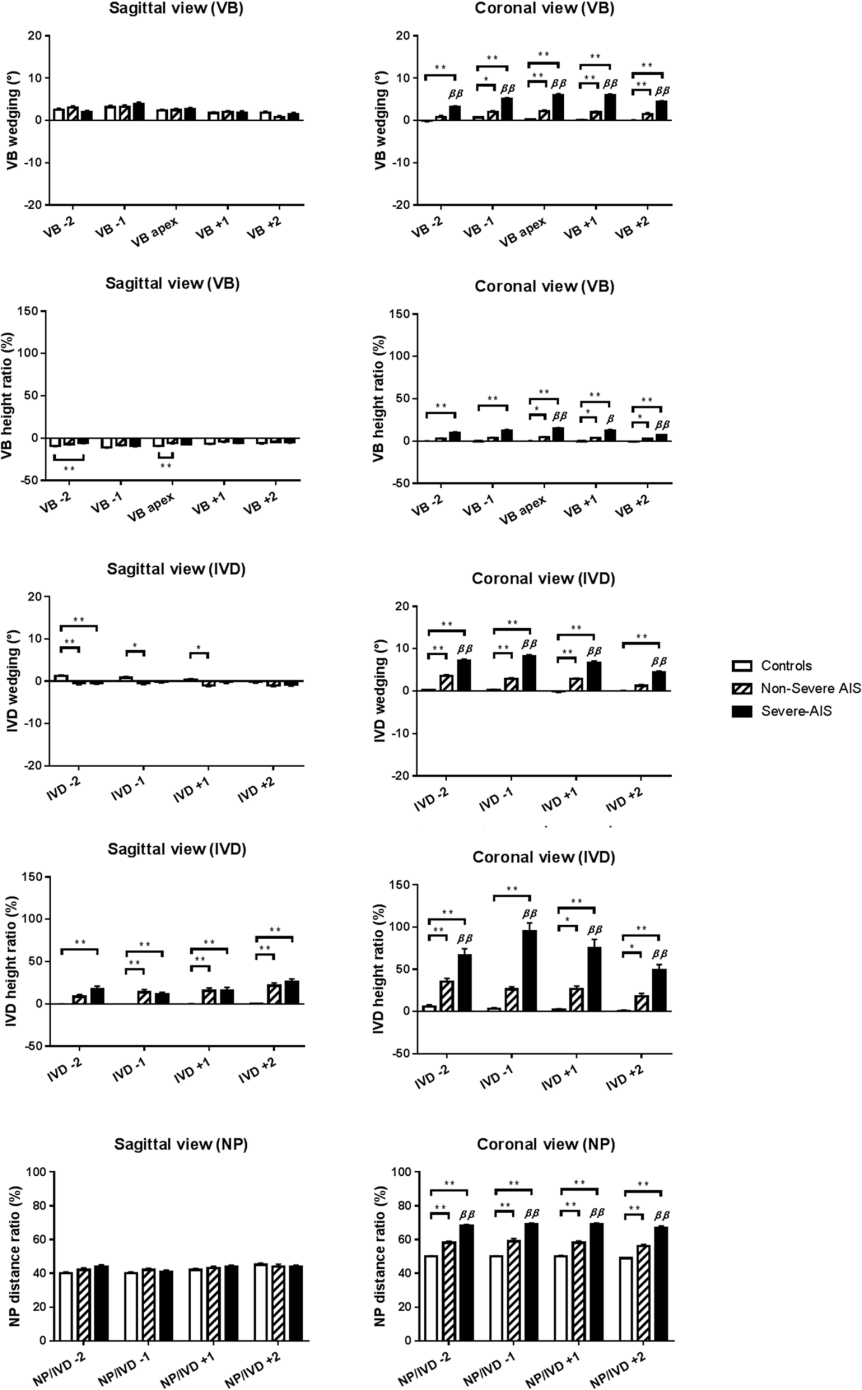
Graphic summary of mean values of different MRI parameters measured at different vertebral and disc levels among healthy controls, non-severe AIS and severe-AIS groups. * *P* < 0.05, ** *P* < 0.01, when comparison with control; ^ß^ *P* < 0.05, ^ßß^ *P* < 0.01, when compared with non-severe AIS; Data expressed as Mean ± Standard error of mean; VB, vertebral body; IVD, intervertebral disc; NP, nucleus pulposus

**Fig. 6:**
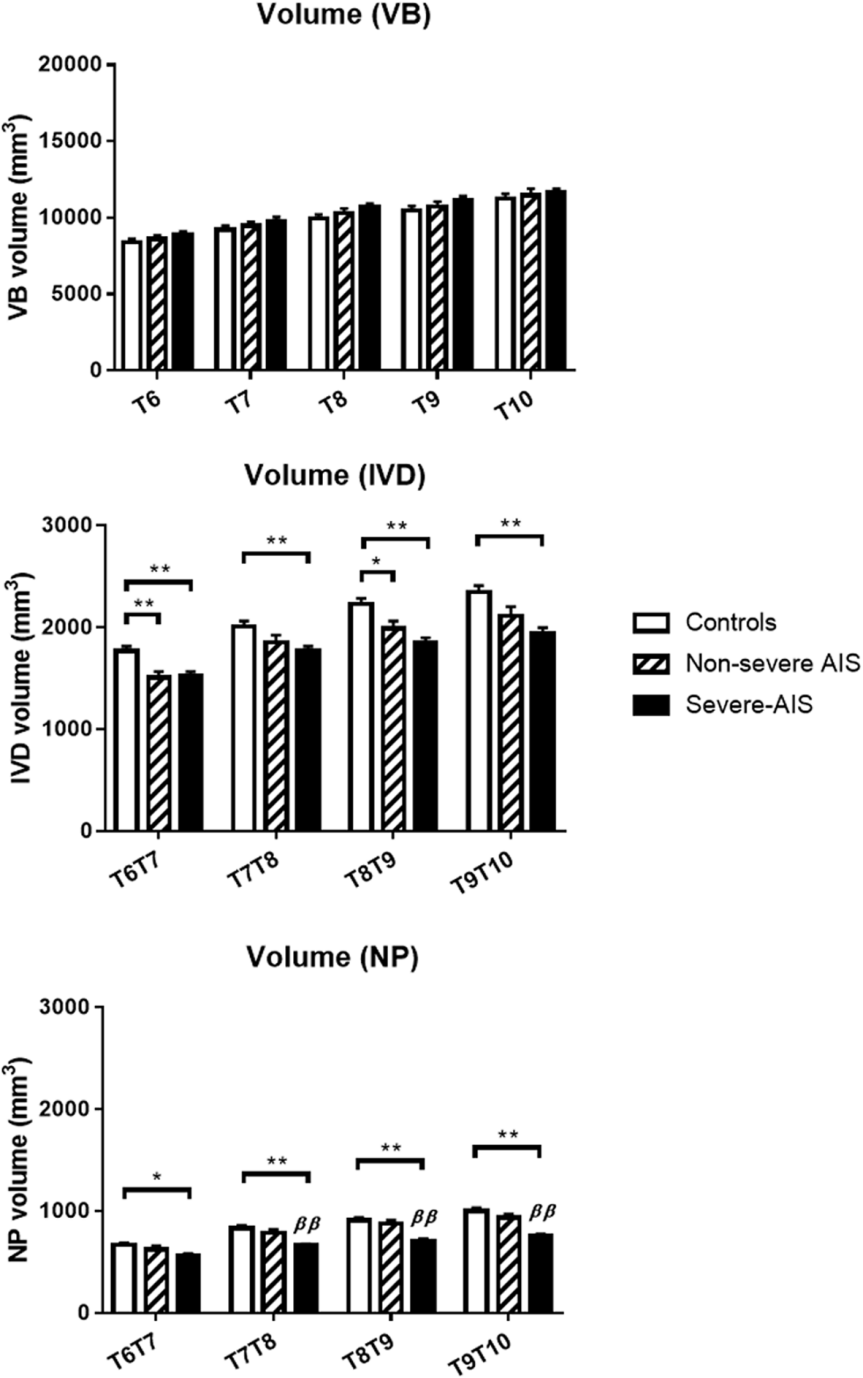
Graphic summary of mean volumetric volumes of vertebral bodies (VB), intervertebral discs (IVD) and nucleus pulposus (NP) measured at different vertebral and disc levels among healthy controls, non-severe AIS and severe-AIS groups. * *P* < 0.05, ** *P* < 0.01, when comparison with control; ^ß^ *P* < 0.05, ^ßß^ *P* < 0.01, when compared with non-severe AIS; Data expressed as Mean ± Standard error of mean

**Fig. 7:**
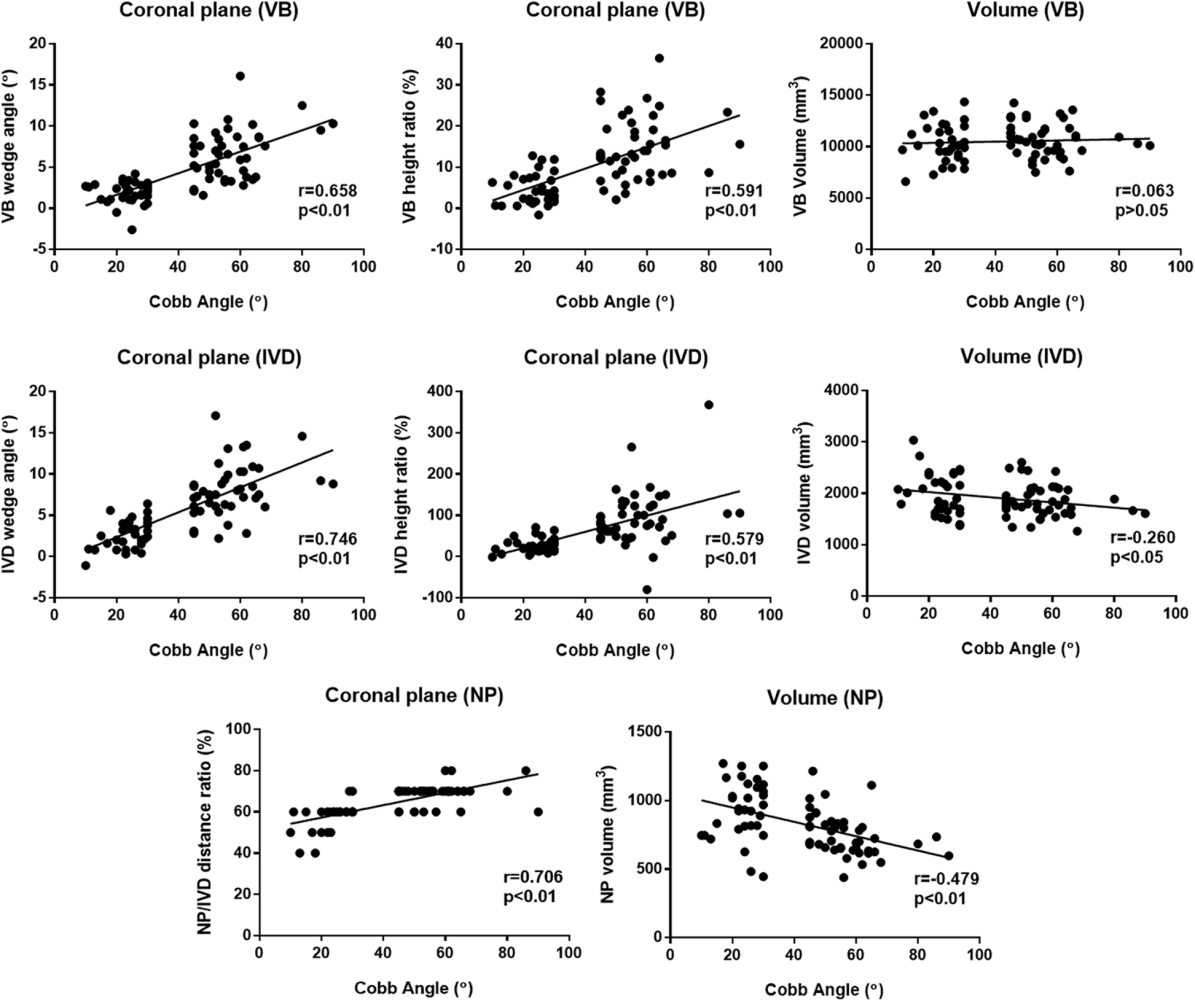
Graphic summary showing relationships between Cobb angles and different MR parameters for AIS patients only. Pearson correlation coefficient (r) and regression lines are shown if there are significant correlations and linear relations. VB, vertebral body; IVD, intervertebral disc; NP, nucleus pulposus

There was a significant difference in some parameters at apex among three groups (Severe-AIS, non-severe AIS and normal controls) as shown in Fig. 5. On the coronal plane, there was a significant higher mean value of VB wedge angle and VB ${C}_{V}{C}_{c}$ height ratio in the non-severe AIS (*P* < 0.001) and severe-AIS groups (*P* < 0.001) at the apical level when compared with controls. There was also a significant higher mean value of both parameters in severe-AIS (*P* < 0.001) than non-severe AIS groups. On the sagittal plane, non-severe AIS group (*P* < 0.01) had significant lower VB AP height ratio than controls. However, no significant difference was found in VB wedge angle and volume (Fig. 6) between three groups (Table 2). In Fig. 7, Cobb angle was found to be significantly correlated with coronal VB wedge angle (*r* = 0.658, *P* < 0.01) and coronal VB ${C}_{V}{C}_{c}$ height ratio (*r* = 0.591, *P* < 0.01). Also, the volume of VB was significantly correlated with age (*r* = 0.399, *P* < 0.01), body weight (*r* = 0.339, *P* < 0.01), height (*r* = 0.600, *P* < 0.01) and arm-span (*r* = 0.478, *P* < 0.01).

### Intervertebral discs (IVD) measurement analysis

Statistical graphs are shown in Figs. 5, 6 and 7. On the coronal plane, a significantly higher mean value of IVD wedge angle and IVD ${C}_{V}{C}_{c}$ height ratio was found in the non-severe AIS (*P* < 0.001) and severe-AIS groups (*P* < 0.001) at the apical level when compared with controls. Further comparison in both parameters between both AIS groups showed that severe-AIS was significantly higher (*P* < 0.001) than non-severe AIS. On the sagittal plane, there was a significant higher mean value of IVD wedge angle in non-severe AIS (*P* < 0.05) than controls. Moreover, significantly higher IVD AP height ratio was found in non-severe AIS (*P* < 0.001) and severe-AIS (*P* < 0.001) groups than controls. The IVD volume was significantly reduced in non-severe AIS (*P* < 0.05) and severe-AIS (*P* < 0.001) groups when compared with controls (Table 3). The Cobb angle was significantly correlated with coronal IVD wedging (*r* = 0.746, *P* < 0.01), coronal IVD height ratio (*r* = 0.579, *P* < 0.01) and IVD volume (*r* = -0.260, *P* < 0.05).

### Nucleus pulposus (NP) measurement analysis

Statistical graphs are shown in Figs. 5, 6 and 7. NP was significantly shifted to the convexity in non-severe AIS (*P* < 0.001) and severe-AIS groups (*P* < 0.001) when compared with controls, i.e. a significant higher NP/IVD distance ratio towards the value of 1 on coronal plane. However, no significant difference for the NP/IVD distance ratio was found on sagittal plane among three groups. The mean values of NP volume and IVD volume were significantly lower in severe-AIS when compared with the controls (*P* < 0.001) and non-severe AIS (*P* < 0.001) groups. Cobb angle was significantly correlated with the coronal NP/IVD distance ratio (*r* = 0.706, *P* < 0.01) and NP volume (*r* = -0.479, *P* < 0.01). The volume of NP was significantly correlated with body weight (*r* = 0.347, *P* < 0.01), height (*r* = 0.219, *P* < 0.05) and BMI (*r* = 0.302, *P* < 0.01).

## Discussion

This study is demonstrating the difference in volumetric IVD measurement in AIS with right-sided thoracic curvature and compared with age- and sex-matched non-AIS asymptomatic controls. The current finding shows that there are significantly larger VB wedge angle and higher VB height ratio on the coronal plane indicating vertebral wedging towards the concavity side of the scoliotic curve in AIS groups. There are significantly larger IVD wedge angle and higher IVD height ratios, indicating more IVD wedging towards the convexity and posterior margin at the apical region of IVD in AIS groups. Whereas, the volume of IVD is significantly lower in AIS groups. In this study, the position of NP inside the IVD is significantly shifted to the convexity, whereas the volume of NP is significantly reduced in severe-AIS. Overall, there is an increasing trend in IVD wedge angle and height ratio on the coronal plane in AIS with increasing severity. Literally, the above association of VB and IVD changes with curve severity is further supported by a significant positive correlation between the Cobb angle and the aforementioned parameters measured on the coronal plane. The significant volume loss of IVD and NP observed in severe-AIS might be explained as a consequence of the spinal deformity.

Current literatures have often heterogenetic cohorts of AIS subjects for investigation and these studies only demonstrated vertebral measurements in imaging using mixed curve severity, mixed curve type or mixed gender [9, 10, 17, 18, 24]. However, there is an inhomogeneity in AIS subjects and different treatment planning should be determined and offered based on their curve magnitude in Cobb angle. These heterogeneities can affect the outcome being reported, owing to the difference in apical location, side and severity of the spinal curvatures in the AIS subjects being used. In addition, these studies lack comparison with asymptomatic controls as a global reference. Hence, our study aimed to aimed to conduct comparison on multiple musculoskeletal parameters in AIS subjects with homogeneity of curve patterns and different severity, in comparison with non-AIS age- and sex-matched asymptomatic controls using MRI. Overall, these research works would be very useful for better understanding of the true nature of this scoliotic disorder, which can be benefited by providing more rational on preventing curve progression in these AIS patients, and comparing with controls as a reference.

Previous literatures have already speculated the contribution of disc in the growth and development of scoliotic deformity [11, 13]. Early studies using conventional X-rays showed the presence of vertebral and intervertebral wedging with an increase in curve severity at both thoracic and lumbar regions [15, 22]. Likewise, a longitudinal study using serial spine radiographs found that the occurrence of disc wedging can led to curve progression during rapid growth spurt of AIS patients [14]. In connection, a previous CT study by our group illustrating the changes of VB and IVD with curve severity in AIS have also complement the above finding [9, 10]. Whereas in the current study, we have further explored the morphology of IVD (including the NP) using MRI, which is known to provide better soft tissue resolution and hence superb morphological delineation of IVD when compared with CT. Furthermore, as MRI is radiation free, we were able to include non-severe AIS and normal healthy subjects in the current study while in previous CT studies, only severe-AIS subjects who underwent pre-op planning were included in view of the high radiation dose of CT examinations. In brief, the findings in the current study concur with other studies using CT [9, 10]. It showed a relative anterior lengthening of the spinal column, which is mainly contributed by the anterior to posterior height difference in IVD on sagittal plane of AIS subjects when compared with controls. The observed uneven anteroposterior disc wedge might be caused by the vertebral compression during progression of the spinal deformity. Our results also show that there was a significant correlation between the IVD wedging with curve severity which are consistent with the literatures [17, 18]. Moreover, there is an increasing trend in VB and IVD wedging when compared between the asymptomatic controls, non-severe AIS and severe-AIS groups.

Previous MRI studies by Périé et al. reported nucleus zone migration to the convexity of the scoliotic curvature, of which correlation between the nucleus zone migration and intervertebral wedging was found using the 3D geometric method [24, 25]. Similarly, Violas et al. also found nucleus migration at the apical level of scoliotic curvature when Cobb angle is greater than 20 degrees [20, 26]. In the same study with AIS patients concerned, volume and hydration of IVD within the lumbar spine before and after surgery were evaluated [20]. An increase in nucleus-disc volume ratio was found post-op, implying rehydration of the subjacent discs after the spinal correction. The above finding has important impacts on timing of optimal surgical strategy. IVD is a complex structure within vertebral column of human. Though disc degeneration is a known process in aging due to biomechanical changes, it is not yet fully understood in AIS whether excessive mechanical stress onto the disc by the spinal deformity might lead to the early disc degeneration when compared with those without scoliosis [27]. Nevertheless, some studies already showed there were greater calcification and less organized elastic fiber network in AIS discs when compared with controls [28, 29].

Our previous studies showed that the presence of increased spinal cord tethering was associated with morphological changes of cross-sectional shape and relative position of the cord involving in development of scoliosis [6, 7]. These features suggested that the relative shortening and functional tethering of spinal cord may play an important role in the etiopathogenesis of AIS. In routine clinical practice, disc degeneration is first depicted by loss of high T2 signal within the disc, which is also known as disc desiccation. However, in the current study and the previous study by Violas et al*.*, it clearly showed that earlier signs of premature degeneration of the NP can be detected before desiccation when using volumetric measurement of NP [20]. If we consider the classical MR features of disc height reduction and loss of T2 signal indicate advanced and irreversible disc degeneration, we postulate that the morphological changes of IVD shown in the current study might represent early, and possibly reversible, degenerative process. Thus, IVD changes in severe-AIS might potentially progress to irreversible disc degeneration if the condition persists or further deteriorates. These patients might sustain from low back pain before their normal elderly age if no operative treatment is offered. Therefore, an early brace treatment is highly recommended for AIS subjects to prevent any curve progression before reaching to severe-AIS with an early disc degeneration [30].

There are a few limitations in this study. Firstly, the sample size is small. In addition, the current study is a cross-sectional study; hence there is no longitudinal data to demonstrate the change in IVD with curve progression. A longitudinal study with a larger cohort to evaluate the serial changes of IVD in AIS subjects with curve progression would be helpful in improving the understanding on the mechanism of disc compression by change of spinal curvature. In addition, future studies across the spectrum of growth could be employed in younger AIS children to evaluate the soft tissues of the unossified vertebral body growth centres and non-osseous soft tissues that cannot be imaged with CT. However, it is expensive and time consuming imaged with MRI. Subject has to be as still as possible during the MRI scan is operated, otherwise, subject motion during the acquisition can induce artefacts and reduce image quality, and diagnostic relevance. Furthermore, a long-term follow-up study comparing early and late surgical correction, and irreversible disc damage correlation should be warranted.

## Conclusions

In summary, this study has demonstrated that in addition to wedging of VB and IVD, there was significantly reduced volume of IVD and NP in AIS patients with severe curve, insinuating the mechanical effect of scoliosis leads to a compression on both IVD and NP before significant disc desiccation occurs. We postulate that the compression of IVD and NP can contribute to curve progression in severe-AIS, in which these patients are more vulnerable of disc degeneration in adulthood. Timely corrective treatment should be offered to avoid irreversible disc damage by progressive spinal deformity. Further longitudinal study on these parameters contributing to curve progression in severe-AIS is still warranted.

**Table 1:** Demographic data of healthy controls and AIS subjects with different curve severities

Parameters	Controls (*n* = 33)	Non-Severe AIS (*n* = 32)	Severe-AIS (*n* = 44)	*P*-value
Age (years)	14.8 ± 0.3	14.1 ± 0.3	14.3 ± 0.3	0.258
Cobb angle (^o^)	–	24.0 ± 1.1	55.6 ± 1.7	< 0.001**
Body weight (kg)	47.9 ± 1.9	44.6 ± 1.3	43.7 ± 1.1	0.101
Body height (cm)	155.8 ± 1.2	156.8 ± 1.1	155.7 ± 1.1	0.762
BMI (kg/m^2^)	19.5 ± 0.6	18.1 ± 0.4	18.0 ± 0.3	0.027*
Armspan (cm)	155.1 ± 1.3	156.0 ± 1.3	156.8 ± 1.3	0.666
BMI with armspan (kg/m^2^)	19.7 ± 0.6	18.3 ± 0.5	17.7 ± 0.3	0.011*

One-way ANOVA with *P*-value * < 0.05, ** < 0.01

Data expressed as Mean ± Standard error of mean

**Table 2:** Group comparison of different MRI parameters among healthy controls, non-severe AIS and severe-AIS subjects

Parameters		Sagittal				Coronal			
		Controls	Non-severe AIS	Severe-AIS	*P*-value	Controls	Non-severe AIS	Severe-AIS	*P*-value
VB	Wedge angle (˚)	2.3 ± 0.3	2.4 ± 0.4	2.6 ± 0.4	0.840	0.2 ± 0.2	2.3 ± 0.3^##^	5.9 ± 0.4^## *ßß*^	< 0.001**
	Height ratio (%)	-9.1 ± 0.5	-5.8 ± 0.8^##^	-7.1 ± 0.7	0.008**	0.0 ± 0.3	4.6 ± 0.7^#^	14.7 ± 1.1^## *ßß*^	< 0.001**
IVD	Wedge angle (˚)	0.3 ± 0.3	-1.0 ± 0.3^#^	-0.2 ± 0.3	0.017*	-0.1 ± 0.2	2.7 ± 0.2^##^	6.6 ± 0.5^## *ßß*^	< 0.001**
	Height ratio (%)	0.1 ± 0.1	15.6 ± 3.4^##^	15.7 ± 3.7^##^	0.001**	1.9 ± 1.2	26.8 ± 3.4^#^	75.1 ± 7.9^## *ßß*^	< 0.001**
NP/ IVD	Distance ratio (%)	42.0 ± 0.9	43.0 ± 1.2	44.0 ± 1.0	0.155	50.0 ± 0.5	58.0 ± 1.2^##^	69.0 ± 0.8^## *ßß*^	< 0.001**

One-way analysis of variance (ANOVA): *P < 0.05, **P < 0.01; Post hoc test (Bonferroni correction test) in comparison with controls: ^#^ *P* < 0.05, ^##^ *P* < 0.01; in comparison with non-severe AIS: ^ß^ *P* < 0.05, ^ßß^ *P* < 0.01;

Data expressed as Mean ± Standard error of mean

*VB* Vertebral body; *IVD* Intervertebral disc; *NP* Nucleus pulposus

**Table 3:** Group comparison of volumetric measurement of vertebral bodies, intervertebral discs and nucleus pulposus among healthy controls, non-severe AIS and severe AIS

Parameters		Controls	Non-severe AIS	Severe-AIS	*P*-value
Volume (mm^3^)	VB	9930 ± 283	10,274 ± 328	10,675 ± 240	0.159
	IVD	2232 ± 55	1992 ± 72^**#**^	1852 ± 47^**##**^	< 0.001**
	NP	911 ± 28	875 ± 38	708 ± 24^**## *ßß***^	< 0.001**

One-way analysis of variance (ANOVA): *P < 0.05, **P < 0.01; Post hoc test (Bonferroni correction test) in comparison with controls: ^#^ *P* < 0.05, ^##^ *P* < 0.01; in comparison with non-severe AIS: ^ß^ *P* < 0.05, ^ßß^ *P* < 0.01;

Data expressed as Mean ± Standard error of mean;

*VB* Vertebral body; *IVD* Intervertebral disc; *NP* Nucleus pulposus

## Data Availability

The datasets used and/or analyzed during the current study are available from the corresponding author on reasonable request.

## Abbreviations

3D: Three-dimensional
AIS: Adolescent idiopathic scoliosis
ANOVA: Analysis of variance
AP: Anterior-posterior
$${C}_{v}{C}_{c}$$: Convexity-concavity
CT: Computed tomography
ICC: Intraclass correlation coefficient
IVD: Intervertebral disc
MRI: Magnetic resonance imaging
NP: Nucleus pulposus
VB: Vertebral body
WA: Wedge angle
